# Successful treatment of recurrent traumatic cardiac arrest due to splenic rupture in trauma resuscitation unit: a case report

**DOI:** 10.3389/fmed.2025.1650387

**Published:** 2025-10-01

**Authors:** Ruonan Gu, Haimin Hu, Haiping Zhao, Yuhua Shen, Hailun Gu, Weijie Han, Gefei Jiang, Shouyin Jiang

**Affiliations:** ^1^Department of Emergency Medicine, Haiyan County People's Hospital, Jiaxing, Zhejiang, China; ^2^Department of Emergency Medicine, Second Affiliated Hospital, Zhejiang University School of Medicine, Hangzhou, China; ^3^Zhejiang Key Laboratory of Trauma, Burn, and Medical Rescue, Hangzhou, China; ^4^Zhejiang Province Clinical Research Center for Emergency and Critical Care Medicine, Hangzhou, China; ^5^Research Institute of Emergency Medicine, Zhejiang University, Hangzhou, China; ^6^National Emergency Medical Rescue base, Hangzhou, China

**Keywords:** traumatic cardiac arrest, splenic rupture, resuscitation, emergency laparotomy, hemorrhagic shock

## Abstract

Recurrent traumatic cardiac arrest (rTCA) represents a critical emergency with mortality rates exceeding 96% and limited neurological recovery among survivors. High-quality evidence to guide standardized management remains scarce. We report the case of a 67-year-old male presenting with rTCA secondary to Grade IV splenic rupture following a high-impact traffic collision. Upon emergency department arrival, the patient experienced cardiac arrest, achieving temporary return of spontaneous circulation before a second arrest 8 min later due to massive intra-abdominal hemorrhage. Focused assessment with sonography for trauma (FAST) and diagnostic paracentesis confirmed splenic rupture as the primary cause. Due to persistent hemodynamic instability precluding transfer, emergency laparotomy was determined 10 min post-arrival and performed in the trauma resuscitation unit (TRU) 25 min latter. Intraoperatively, 2,500 mL of blood and 300 g of clots were evacuated, followed by splenectomy for definitive hemostasis. Aggressive resuscitation, including tranexamic acid, prothrombin complex concentrate, and packed red blood cell transfusion within 23 min, alongside multidisciplinary intensive care, facilitated hemodynamic stabilization within 48 h. Full neurological recovery was evident by day 5, with successful extubation on day 7 and discharge on day 25 with restored functional independence. This case highlights three key principles for rTCA management: (1) surgical strategy (laparotomy versus thoracotomy) and venue (TRU versus operating room) must be informed by integrated injury assessment and hemodynamic status; (2) early hemostatic control is critical to interrupting recurrent arrests; and (3) multimodal, goal-directed resuscitation and interdisciplinary collaboration are essential for optimizing survival and neurological outcomes. These insights offer a robust framework for trauma teams managing complex rTCA cases.

## Introduction

Traumatic cardiac arrest (TCA) remains a formidable challenge in emergency medicine, characterized by a grave prognosis despite significant advancements in resuscitative strategies. Current literature underscores the dismal outcomes associated with TCA, with an overall mortality rate exceeding 96% and only 43.5% of survivors achieving favorable neurological recovery (1). Effective management of TCA necessitates rapid, evidence-based decision-making, particularly regarding the choice of surgical intervention (thoracotomy versus laparotomy) and the optimal operative setting (operating room [OR] versus trauma resuscitation unit [TRU]). Recurrent traumatic cardiac arrest (rTCA), defined as repeated cardiac arrest following initial return of spontaneous circulation (ROSC) in trauma patients, often results from unaddressed reversible causes, such as hemorrhage from solid organ or vascular injuries (1). However, high-quality evidence to guide these critical decisions, especially in rTCA, remains limited, contributing to significant variability in clinical practice.

In cases of thoracoabdominal trauma, the initial therapeutic dilemma centers on prioritizing thoracotomy or laparotomy. Thoracotomy facilitates direct cardiac access, aortic cross-clamping to enhance cardio-cerebral perfusion, and intracardiac massage, whereas laparotomy focuses on controlling intra-abdominal hemorrhage (2, 3). This decision is complicated by the absence of standardized guidelines and the heterogeneity of clinical presentations. Similarly, the choice of operative venue is critical. While the OR has traditionally been the preferred setting for definitive trauma surgery, TRU-based interventions may offer survival benefits for patients with refractory hemorrhagic shock too unstable for transfer (4). Elucidating the factors influencing these decisions is essential for optimizing patient outcomes.

Successful extubation and neurological recovery in TCA patients are exceedingly rare, making the identification of factors contributing to these outcomes critical for refining resuscitative protocols and improving prognostic accuracy. Through a detailed analysis of a complex clinical case and a comprehensive review of the existing literature, this report aims to address these knowledge gaps and provide actionable insights for multidisciplinary trauma teams. This case report adheres to the SCARE 2023 Criteria for standardized reporting (5).

## Case presentation

A 67-year-old male (height: 170 cm, weight: 75 kg) presented to the emergency resuscitation unit following 30 min of unconsciousness after a traffic collision. The patient sustained blunt thoracoabdominal trauma after colliding with a roadside guardrail while riding an electric vehicle, followed by a fall. Emergency medical services (EMS) were contacted by bystanders, and the patient was transported to the emergency department (ED). On arrival, he was unresponsive, pale, with cold, clammy extremities. Three minutes post-arrival, blood pressure became unmeasurable, large artery pulsations ceased, and cardiac and respiratory arrest ensued. High-quality cardiopulmonary resuscitation (CPR) and advanced cardiac life support (ACLS) were immediately initiated. His medical history included hypertension, diabetes mellitus, and a prior intestinal tumor, with no known allergies. He was a non-smoker with a 40-year history of alcohol consumption.

**Figure 1:**
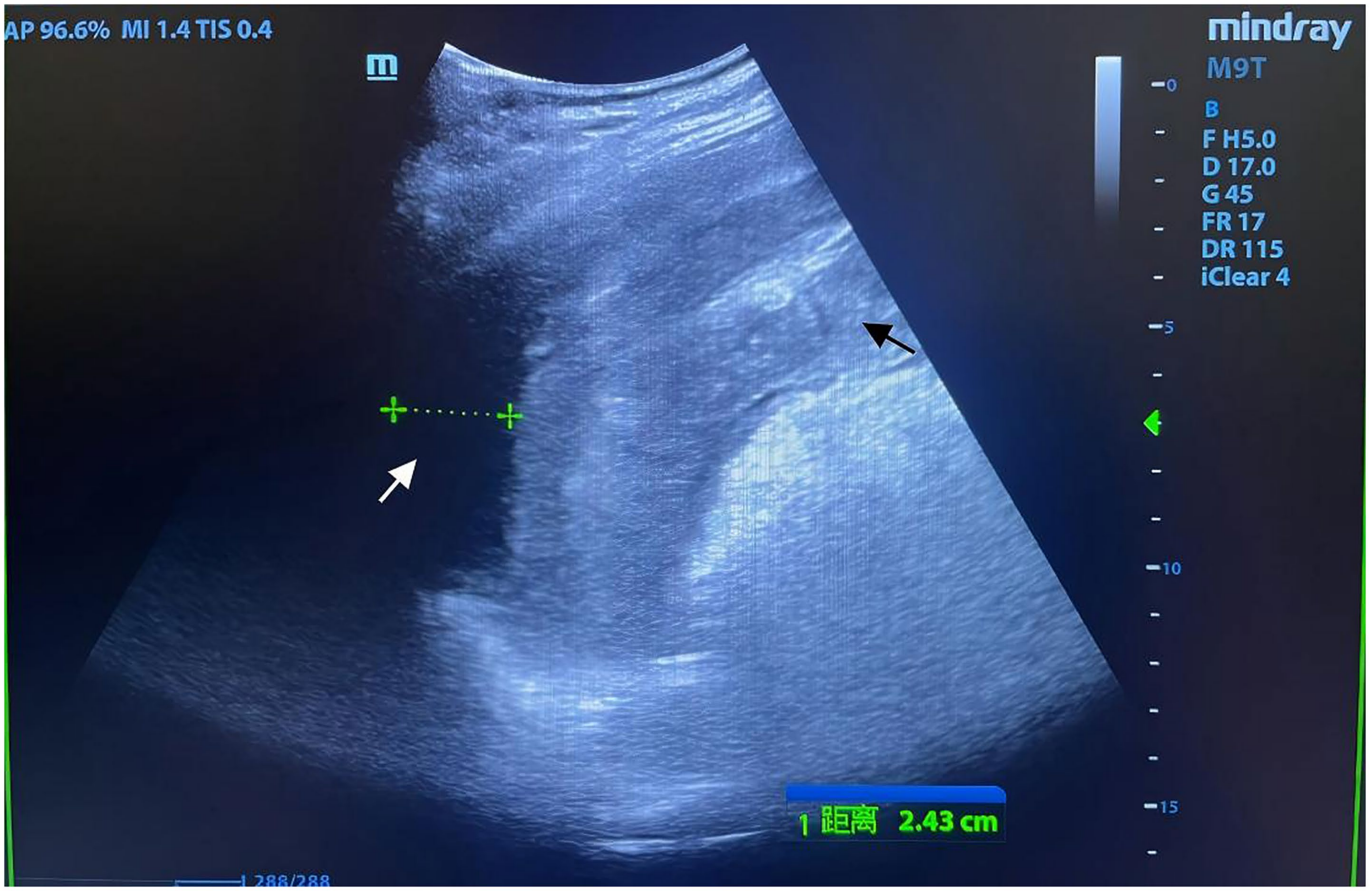
Focused assessment with sonography for trauma (FAST) imaging during emergency resuscitation. Rapid-sequence FAST performed during urgent resuscitation revealed an indistinct splenic contour with irregular morphology and heterogeneous echotexture (black arrow). A hypoechoic perisplenic fluid collection, measuring 24 mm in width with poor acoustic transmission, was identified (white arrow). Intraoperative findings confirmed that the volume of intra-abdominal hemorrhage exceeded the extent visualized on FAST, correlating with the patient’s refractory hemorrhagic shock.

**Figure 2:**
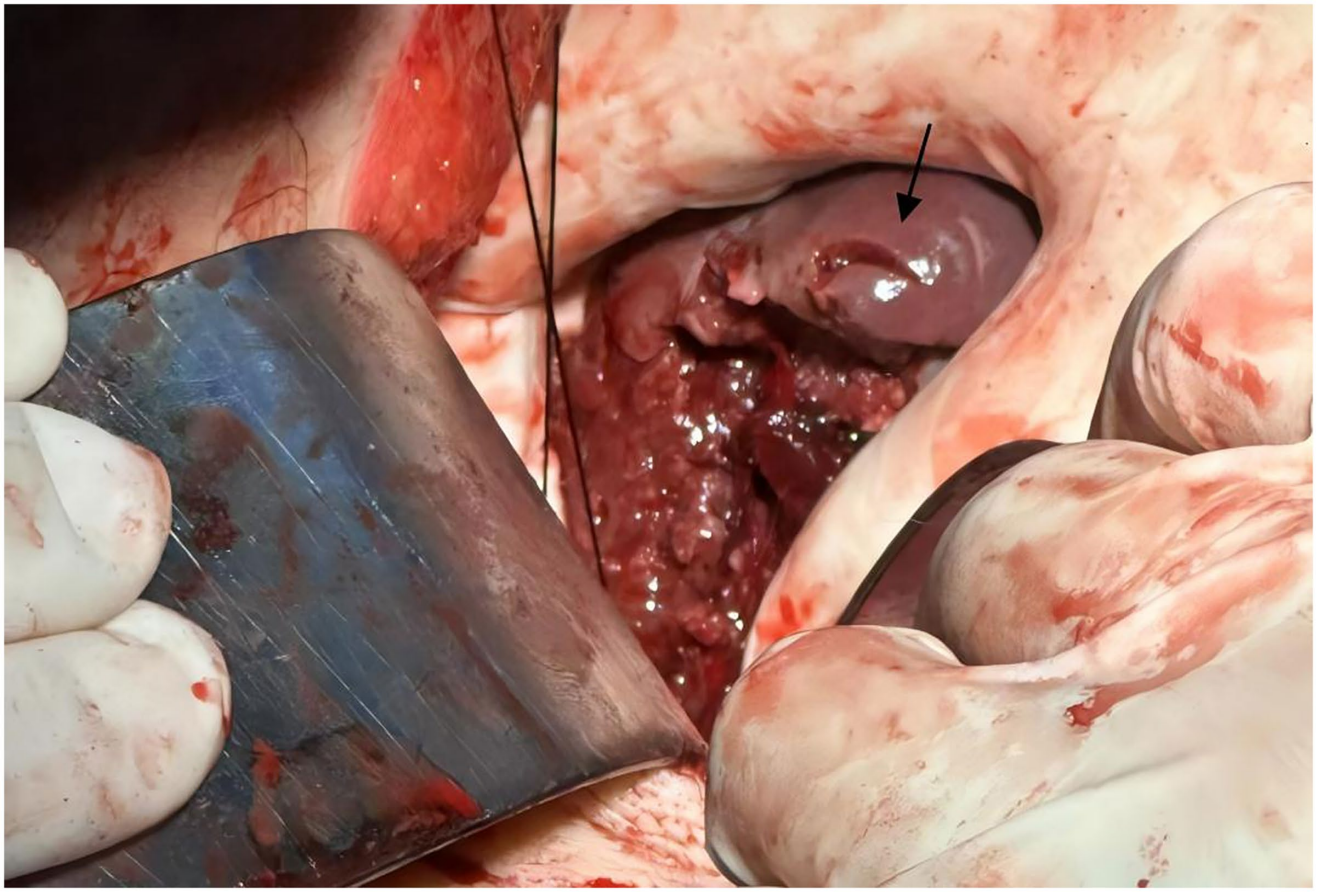
Intraoperative image of splenic rupture. Intraoperative photograph obtained during emergency laparotomy, depicting a partial splenic laceration (black arrow). Image quality reflects limitations due to the urgent resuscitative context.

Physical examination revealed a 14-cm open wound on the left chest wall without thoracic cavity penetration and abdominal distension with dullness on percussion. During CPR, emergency trauma physicians performed a focused assessment with sonography in trauma (FAST), with the multidisciplinary team arriving promptly. FAST revealed significant intra-abdominal fluid and signs of splenic rupture (Figure 1), with no evidence of massive hemothorax or pericardial effusion in the thoracic cavity. Diagnostic abdominal paracentesis yielded non-clotting blood, strongly indicating traumatic splenic rupture with hemorrhagic shock as the primary cause of cardiac arrest. Due to profound hemodynamic instability, recurrent cardiac arrests, and inability to tolerate transport, emergency laparotomy was planned 10 min post-arrival and performed in the TRU. Within 12 min of ED arrival, tranexamic acid, albumin, and prothrombin complex concentrate were administered, and requests for packed red blood cells (PRBCs) and plasma were initiated. Central venous catheter (CVC) access was established. ROSC was achieved 13 min post-arrival; however, a second cardiac arrest occurred at 21 min. By 23 min, PRBC transfusion was initiated. ROSC was re-established prior to surgical incision. At 35 min post-arrival, an experienced trauma surgical team commenced emergency laparotomy in the TRU. Upon entering the abdominal cavity, approximately 2,500 mL of blood and 300 g of blood clots were aspirated. Severe splenic rupture was confirmed, with multiple longitudinal tears in the oblique dorsal capsule of the upper and middle splenic segments, the longest measuring 5 cm (Figure 2). Per the American Association for the Surgery of Trauma (AAST) criteria, the splenic rupture was classified as Grade IV. The splenic artery and vein were clamped for hemostasis, followed by splenectomy. Thoracic surgeons subsequently performed debridement and suturing of the chest wall wound and placed a chest drain.

Laboratory findings indicated acute blood loss, coagulopathy, and shock: hemoglobin 89 g/L, platelet count 36 × 10^9^/L, prothrombin time 15 s, thrombin time 26.9 s, fibrinogen 1.36 g/L, activated partial thromboplastin time 32.2 s, and international normalized ratio 1.31. Arterial blood gas analysis revealed pH 7.09, lactate 14.7 mmol/L, and base excess −14.9 mmol/L. Surgery concluded 2 h and 13 min post-ED arrival. Immediate postoperative computed tomography (CT) of the head, cervical spine, chest, and abdomen revealed bilateral pulmonary contusions with infiltrates and partial consolidation in both lower lobes, minimal left traumatic pneumothorax with an indwelling chest tube, small bilateral pleural effusions, calcifications in multiple hilar and mediastinal lymph nodes, multiple bilateral rib fractures, postsplenectomy changes with perisurgical hematoma, minimal pneumoperitoneum, an indwelling abdominal drain, and small-volume fluid collections in the abdomen and pelvis. The Injury Severity Score (ISS) was 34. Thirty-four minutes post-surgery, the patient was transferred to the Trauma intensive care unit (TICU) for intensive monitoring and multidisciplinary management. During surgery and the initial 24 h, the patient received norepinephrine (maximum 2.22 μg/kg/min) and epinephrine (maximum 0.27 μg/kg/min) via micro-pump infusion, alongside permissive fluid resuscitation with crystalloids and colloids, 14 units of PRBCs, 2,660 mL of fresh frozen plasma, 20 IU of cryoprecipitate, 5 g of human fibrinogen, 800 IU of human prothrombin complex concentrate, and 60 g of human albumin. Imipenem-cilastatin was initiated for antimicrobial prophylaxis.

**Figure 3:**
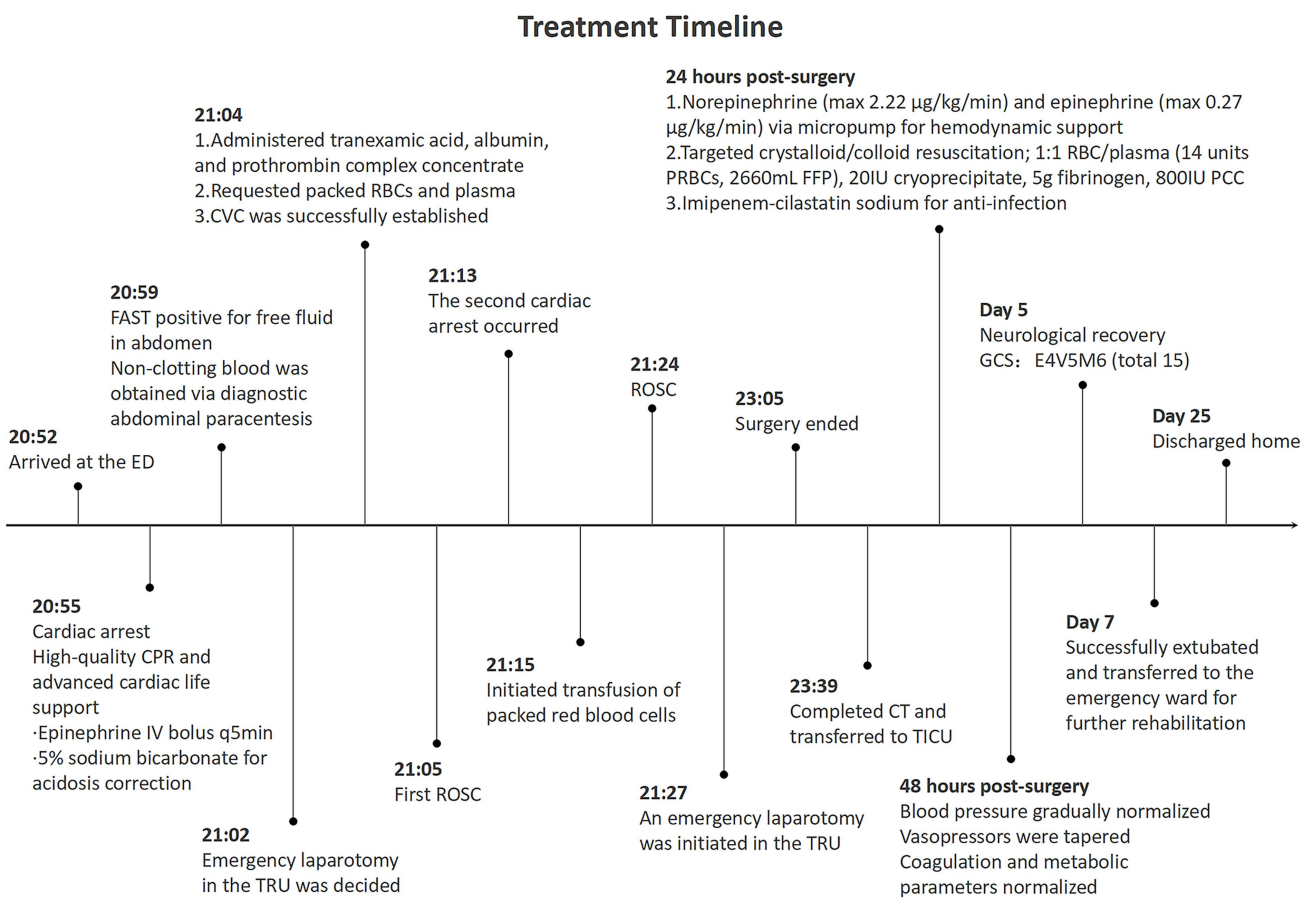
Timeline and key medical events in the management of traumatic cardiac arrest: from emergency department arrival to hospital discharge.

Over the subsequent 48 h, blood pressure stabilized with gradual vasopressor tapering. Hemoglobin stabilized at approximately 110 g/L, platelet count increased to 382 × 10^9^/L, and coagulation and metabolic parameters normalized. On hospital day 5, neurological recovery was evident, with spontaneous eye opening, purposeful motor responses, a Glasgow Coma Scale (GCS) score of 15 (E4V5M6), and neuron-specific enolase levels within normal limits (13.64 ng/mL on hospital day 2 and 10.33 ng/mL on hospital day 3). On day 7, following comprehensive respiratory assessment, the patient was successfully extubated and transferred to the emergency ward for rehabilitation. During rehabilitation, functional recovery progressed steadily, with satisfactory wound healing and restoration of independent ambulation. The patient reported no traumatic stress reactions, and pain was managed within tolerable limits with routine analgesics. At discharge on day 25, the patient expressed satisfaction with rehabilitation outcomes, having resumed daily activities such as self-care and short-distance walking. A detailed treatment timeline (Figure 3) illustrates the clinical course, key interventions, and milestones from ED presentation to discharge, enhancing chronological clarity for clinical reference.

## Discussion

This case of a 67-year-old male surviving TCA secondary to thoracoabdominal blunt trauma underscores the pivotal role of rapid, evidence-based decision-making in optimizing outcomes. The patient’s survival was driven by the timely selection of laparotomy over thoracotomy, the strategic decision to perform surgery in the TRU rather than the OR, and the implementation of aggressive, multidisciplinary resuscitation strategies. These findings highlight the necessity of integrating multiple interdependent factors to guide effective management of complex TCA cases.

The decision between thoracotomy and laparotomy in patients with thoracoabdominal trauma hinges on a comprehensive evaluation of injury patterns. Point-of-care ultrasound (POCUS) is indispensable for assessing thoracic and abdominal pathologies. Thoracotomy is prioritized in cases of tension pneumothorax, massive hemothorax, or cardiac tamponade (2). In this case, normal thoracic POCUS findings, coupled with abdominal POCUS and diagnostic paracentesis revealing non-clotting blood indicative of massive intra-abdominal hemorrhage, confirmed that intra-abdominal organ injury was the primary driver of TCA, justifying the choice of laparotomy.

The mechanism of injury provides critical diagnostic insights. The patient’s unprotected abdominal wall, subjected to high-impact blunt trauma, predisposed the spleen to injury, a common cause of life-threatening intraperitoneal hemorrhage leading to TCA (6–8). In such scenarios, damage control surgery to achieve rapid hemostasis is paramount. The brevity of cardiac arrest is a key determinant; when arrest is brief and intra-abdominal hemorrhage is suspected, laparotomy can expedite hemostasis and circulatory restoration.

Resuscitative endovascular balloon occlusion of the aorta (REBOA) represents a valuable adjunct for managing non-compressible TCA by controlling hemorrhage and augmenting cardio-cerebral perfusion (9). However, its use carries risks, including distal embolism, lower limb ischemia, and multi-organ dysfunction (10). In this case, REBOA was considered given the patient’s traumatic hemorrhagic shock but was deemed unnecessary following ROSC prior to surgery. The risk of distal ischemia outweighed potential benefits in a patient with stabilized circulation, supporting the decision to prioritize urgent laparotomy for definitive splenic hemorrhage control. Nonetheless, REBOA remains a critical preoperative adjunct in scenarios of refractory hemorrhagic shock, particularly when surgical source control is delayed, warranting its consideration in clinical practice.

Resource availability and multidisciplinary expertise are crucial for optimal TCA management. Trauma centers with surgeons proficient in both thoracotomy and laparotomy are best equipped to make evidence-based decisions. Dynamic FAST during resuscitation guides initial surgical strategies (11, 12), minimizing delays associated with computed tomography (13). For patients with refractory hemorrhagic shock, the choice of surgical venue-TRU versus OR-can be outcome-determinative. TRU-based interventions reduce time-to-intervention while maintaining comparable complication rates to OR surgery (14). Given the narrow therapeutic window in TCA, delays increase mortality risk (15, 16). In this case, the patient’s hemodynamic instability and recurrent TCA precluded OR transfer. TRU-based laparotomy enabled immediate control of splenic hemorrhage, complemented by a rapid transfusion protocol that minimized resuscitation times, both critical to survival. Modern TRUs, equipped with advanced resuscitation technologies and staffed by skilled specialists, provide care comparable to OR settings. Venue selection should balance patient acuity, estimated transport time, and procedural complexity, with TRU surgery serving as a salvage option for hemodynamically unstable patients and OR transfer reserved for stable patients requiring complex interventions.

Multiple interrelated factors contributed to the favorable outcome in this case. Early definitive control of the bleeding source addressed the underlying cause of TCA and subsequent organ hypoperfusion (17). Timely laparotomy effectively halted massive splenic hemorrhage, a prerequisite for survival. Aggressive damage control resuscitation, incorporating balanced administration of crystalloids, colloids, packed red blood cells, and fresh frozen plasma, mitigated the lethal triad of acidosis, hypothermia, and coagulopathy (18, 19). Specific interventions, including tranexamic acid, and prothrombin complex concentrate administered within 12 min of arrival, and initiation of packed red blood cell transfusion at 23 min, exemplify this approach. When available, autologous blood salvage systems further bolstered circulatory support. Comprehensive intensive care unit (ICU) management, including advanced monitoring and interventions such as continuous renal replacement therapy and extracorporeal membrane oxygenation, addressed multi-organ dysfunction. The patient’s well-controlled pre-injury comorbidities (hypertension and diabetes mellitus) conferred a physiological reserve that likely enhanced tolerance of aggressive interventions, underscoring the importance of baseline health status. The seamless coordination of emergency physicians, trauma surgeons, anesthesiologists, and ICU nurses exemplifies the critical role of multidisciplinary collaboration.

**Figure 4:**
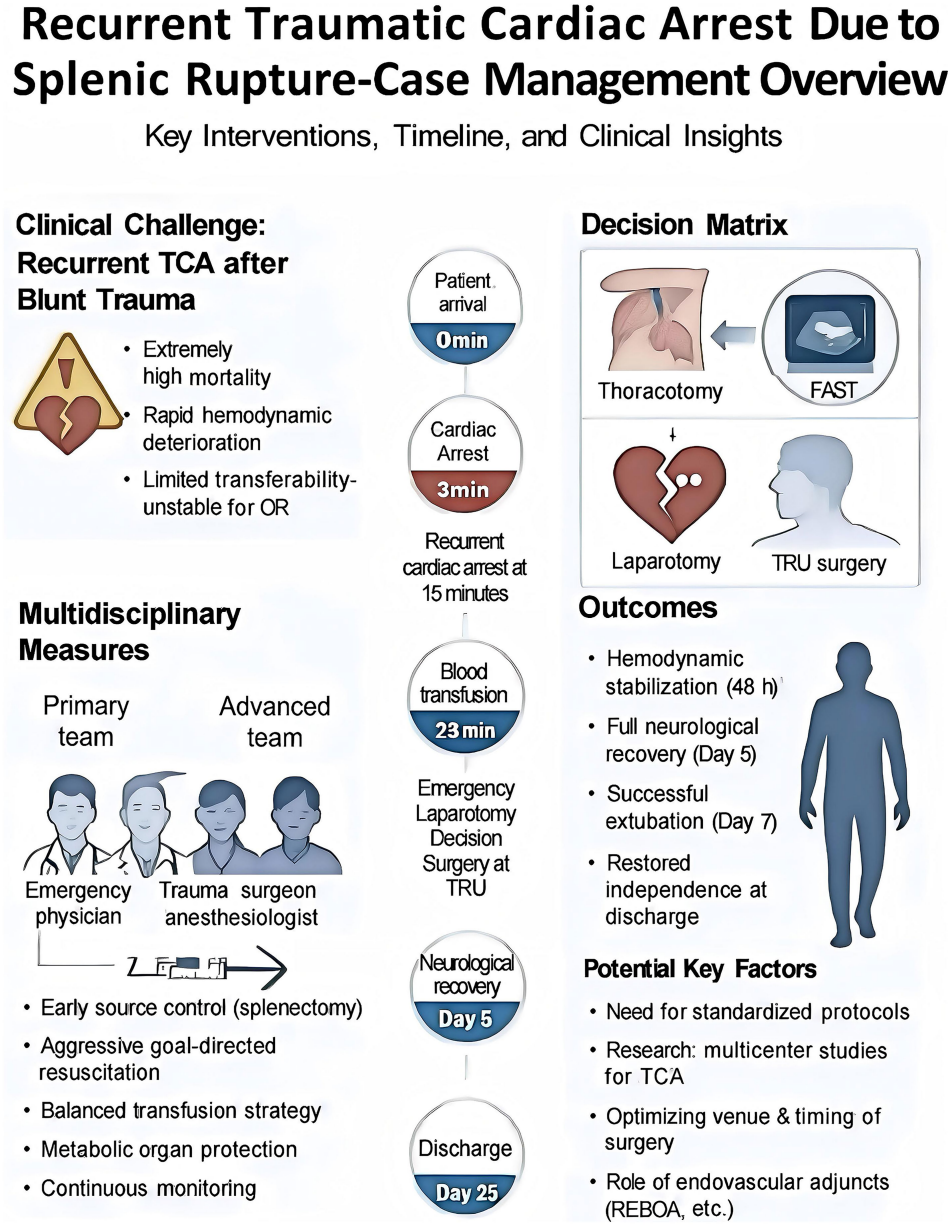
Multidisciplinary management of recurrent traumatic cardiac arrest (rTCA) secondary to splenic rupture: timeline, interventions, and clinical insights. It provides a comprehensive visual representation of the clinical course, interdisciplinary team dynamics, decision-making algorithms, and outcomes for a patient with rTCA due to splenic rupture. It delineates the timeline from initial presentation (0 min) to hospital discharge (Day 25), illustrating pivotal interventions, including resuscitation, blood transfusion, and surgical management. It delineates the roles of primary and advanced multidisciplinary teams, decision matrices guiding surgical approach selection, and key clinical outcomes, such as hemodynamic stabilization, neurological recovery, and successful extubation. The figure underscores the complexities of managing TCA in the context of blunt trauma, highlighting critical multidisciplinary strategies, including early source control via splenectomy, aggressive goal-directed resuscitation, and a balanced transfusion protocol. Additionally, the figure offers insights into the development of standardized protocols for complex trauma care, emphasizing the need for multicenter studies, optimization of surgical timing and venue, and the potential role of endovascular adjuncts in enhancing patient outcomes.

This case provides actionable insights into TCA management, aligning with the principles outlined in Figure 4. First, rapid identification of the bleeding source via FAST for splenic rupture supports integrated injury assessment to guide surgical strategy. Second, early hemostatic interventions, including administration of tranexamic acid, albumin, and prothrombin complex within 12 min and packed red blood cell transfusion at 23 min, exemplify timely, definitive hemostasis critical to interrupting recurrent arrest. Third, real-time monitoring of coagulation, vital signs, and hemodynamics facilitated multimodal, goal-directed resuscitation, prioritizing coagulation stability over volume-based approaches to prevent exacerbation of bleeding. Finally, multidisciplinary collaboration, exemplified by the rapid completion of FAST, hemostatic initiation, and preoperative preparation within 35 min, ensured seamless care delivery. These elements provide a robust framework for trauma teams managing complex recurrent TCA cases, emphasizing rapid, evidence-based interventions and interdisciplinary coordination to optimize survival and neurological outcomes.

### Limitations

This single-case report has inherent limitations in generalizability, as its findings may not be broadly applicable to all patients with rTCA secondary to splenic rupture. Variability in patient characteristics, including age, comorbidities, and injury severity, as well as differences in clinical settings and institutional resources, may influence outcomes. Larger, multicenter studies are warranted to validate these observations and establish broader applicability of the proposed management framework.

## Conclusion

This case elucidates fundamental principles in the management of TCA. The choice between thoracotomy and laparotomy should be informed by a thorough assessment of injury patterns, mechanisms of trauma, and institutional expertise. Emergency TRU surgery represents a critical, life-saving intervention for patients with refractory hemorrhage deemed too unstable for transfer to the operating room. Optimal outcomes hinge on prompt hemostatic control, goal-directed resuscitation, meticulous ICU monitoring, and the patient’s baseline physiological reserve. By synthesizing clinical expertise with evidence-based practice, this report provides a robust framework for trauma teams navigating complex TCA cases, underscoring the critical role of rapid decision-making and interdisciplinary collaboration in enhancing survival and neurological recovery. Notably, the 67-year-old patient’s well-controlled pre-injury comorbidities likely contributed to their ability to withstand aggressive resuscitation and surgical intervention. This observation highlights the importance of evaluating pre-injury physiological reserve when tailoring treatment strategies for similar cases.

## Data Availability

The original contributions presented in the study are included in the article/supplementary material, further inquiries can be directed to the corresponding author.
